# Can NT-pro BNP Levels Predict Prognosis of Patients with Acute Exacerbations of Chronic Obstructive Pulmonary Disease in the Intensive Care Unit?

**DOI:** 10.4274/balkanmedj.2018.0006

**Published:** 2018-11-15

**Authors:** Özlem Ediboğlu, Cenk Kıraklı

**Affiliations:** 1 Intensive Care Unit, University of Health Sciences, İzmir Dr. Suat Seren Chest Diseases and Surgery Training and Research Hospital, İzmir, Turkey

**Keywords:** Chronic obstructive pulmonary disease, mortality, NT-pro BNP

## Abstract

**Background:**

The prognostic value of amino terminal pro-brain natriuretic peptide levels in patients with acute exacerbation of chronic obstructive pulmonary disease has not been fully established.

**Aims:**

To investigate the predictive value of amino terminal pro-brain natriuretic peptide levels in terms of mortality, need for noninvasive mechanical ventilation, invasive mechanical ventilation, and weaning success.

**Study Design:**

Cohort study.

**Methods:**

Patients who were admitted to intensive care unit between December 2015 and December 2016 due to acute exacerbation of chronic obstructive pulmonary disease were included in the study. Demographic data, noninvasive mechanical ventilation application, need for invasive mechanical ventilation, amino terminal pro-brain natriuretic peptide level, duration of mechanical ventilation, intensive care unit and hospital stay, weaning success, and mortality rates were recorded.

**Results:**

A total of 110 patients (75 males) were included in the study. The mean age of the participants was 69 (61-76) years, and the mean Acute Physiology and Chronic Health Evaluation II score was 19 (15-23). The mean amino terminal pro-brain natriuretic peptide level was found to be lower in cases with noninvasive mechanical ventilation success than those with noninvasive mechanical ventilation failure (p=0.053). In addition, the mean amino terminal pro-brain natriuretic peptide level was significantly higher (4740 pg/mL vs. 3004 pg/mL, p=0.001) in patients who needed invasive mechanical ventilation support than in patients who did not. The mortality rate was significantly higher in patients who had an increasing trend of amino terminal pro-brain natriuretic peptide levels during hospitalization than in patients who had decreasing levels (59% vs. 23%, p=0.015). Based on the receiver operating characteristic analysis, the increasing trend of amino terminal pro-brain natriuretic peptide levels during intensive care unit stay predicted mortality with area under curve of 0.84 (p<0.0001, 95% CI: 0.75-0.93) and predicted invasive mechanical ventilation need with area under curve of 0.68.

**Conclusion:**

In cases of acute exacerbation of chronic obstructive pulmonary disease requiring mechanical ventilation, amino terminal pro-brain natriuretic peptide measurement and monitoring of its trend may be a valuable asset in predicting mortality, noninvasive mechanical ventilation, weaning success, and need for invasive mechanical ventilation.

Chronic obstructive pulmonary disease (COPD) is one of the leading causes of morbidity and mortality worldwide. According to Global Initiative for Obstructive Lung Disease 2017, acute exacerbation of COPD (AECOPD) is defined as “acute worsening of respiratory symptoms that needs additional therapies” (1). Many patients require admission to an intensive care unit (ICU), and a substantial percentage (26%-74%) of patients need mechanical ventilatory support (2) due to hypoxemic or hypercapnic respiratory failure. Viral and/or bacterial infections are the leading causes of AECOPD; nevertheless, up to one-third of these cases have an unknown etiology. Cardiovascular diseases are the most common comorbidity in patients with COPD and affect up to 30% of the patients. These diseases are related to increased mortality and are consequently considered as important prognostic factors (3,4). Cardiovascular examination, particularly echocardiography, is essential for diagnosis, follow-up, and treatment of cardiac failures. Although echocardiographic evaluation is an important diagnostic tool for cardiac failure, it has various restrictions, such as imaging difficulty and user dependency during mechanical ventilatory in ICU. Natriuretic peptides, namely, brain natriuretic peptide (BNP) and amino terminal pro BNP (NT-pro BNP), are biomarkers released from ventricles in response to myocardial wall stress in case of impaired myocardial function. These peptides may be used as valid biomarkers for diagnosis of cardiac failure (4,5). Increased BNP levels are associated with several conditions, such as primary pulmonary hypertension, myocarditis, cardiac allograft rejection, right ventricle failure, renal failure, advancing age, and sepsis (6). The right ventricle is mostly affected in COPD, whereas both ventricles may be affected in AECOPD. Hence, high levels of BNP and NT-pro BNP have a prognostic value and are related to increased mortality in AECOPD (7). We aimed to investigate the relationship between NT-pro BNP levels and severity of respiratory failure and outcomes such as noninvasive mechanical ventilatory success, invasive mechanical ventilatory duration, weaning success, and mortality in AECOPD.

## MATERIALS AND METHODS

**Figure 1:**
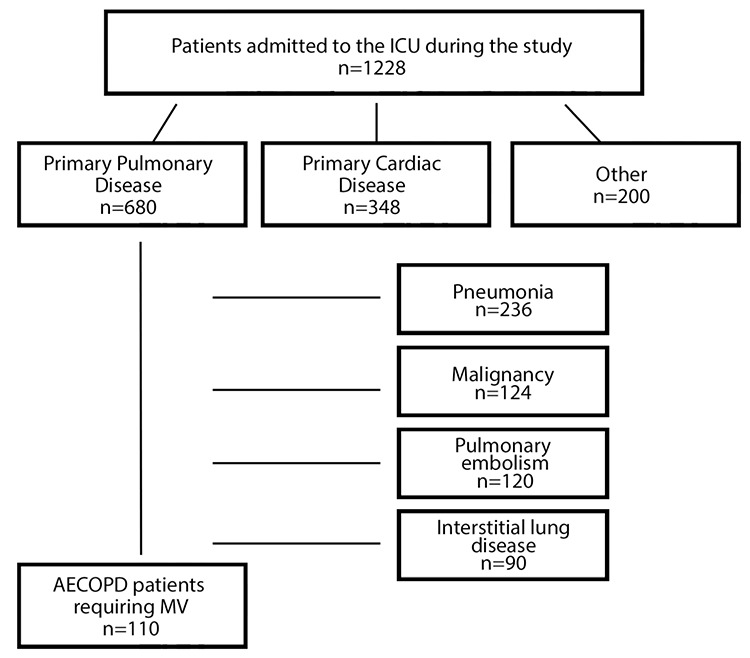
Patient selection criteria. *ICU: intensive care unit; MV: mechanical ventilation*

This retrospective cohort study was performed in a medical ICU of a thoracic disease and surgery training hospital, which is the largest reference center in western Turkey. Schematic of patient selection is shown in Figure 1. Patients who were admitted to ICU, diagnosed with AECOPD due to hypercapnic respiratory failure, and required mechanical ventilatory were included in the study between December 2015 and December 2016. Written informed consent was obtained from the patient. Noninvasive mechanical ventilatory was applied with ICU ventilator (Galileo, Hamilton Medical, Bonaduz, Switzerland) in noninvasive mechanical ventilatory mode via oronasal mask to facilitate respiratory muscle resting in patients with severe dyspnea and tachypnea. Noninvasive mechanical ventilatory application decision was made according to ABG values, such as pH <7.35, pCO_2_ >45 mmHg, and paO_2_/FiO_2_ <200. ABG analysis was performed 2 and 4 hours after the initiation of noninvasive mechanical ventilatory treatment, at least four times a day. Improvement in dyspnea and alertness, decrease in heart and respiratory rate and PaCO_2_, increase in pH, rise of SaO_2_ to 85% or above indicated noninvasive mechanical ventilatory success. Patients who had contraindications for noninvasive mechanical ventilatory application, such as cardiac arrest or dysrhythmias, hemodynamic instability (systolic blood pressure <90 mm Hg), immediate endotracheal intubation necessity, apnea, upper airway obstruction, decreased level of consciousness (moderately severe to severe), upper gastrointestinal bleeding, facial trauma, and vomiting and those who had noninvasive mechanical ventilatory failure were immediately intubated. Invasive mechanical ventilatory was applied through an ICU ventilator in Adaptive Supportive Ventilation mode (Galileo, Hamilton Medical, Bonaduz, Switzerland). Demographic data, such as age, gender, comorbidity, and Acute Physiology and Chronic Health Evaluation (APACHE) II score, and ICU outcomes, such as noninvasive mechanical ventilatory success and need for invasive mechanical ventilatory were analyzed. Weaning was performed according to European Respiratory Society Task Force Criteria (8). Serum levels of NT-pro BNP were measured by electrochemiluminescence method with a moduler Cobas e 411 device (Roche Diagnostics GmbH, D- 68298 Mannheim, Germany). Levels of NT-pro BNP were measured randomly during hospitalization. Trends of serum NT-pro BNP levels during ICU stay were also recorded. The relationship between the trend and outcomes was evaluated. Duration of ICU and hospital stay, weaning, and mortality rate were recorded. Approval was obtained from the Ethics Committee for Non-Drug Investigations in December 5, 2017 (No: 8167).

### Statistical analysis

Statistical analyses were conducted using the SPSS program (Statistical Package for the Social Sciences 21.0 version IBM Corp.; NY, USA). Continuous variables were indicated as median (25-75 percentiles) and compared through Mann-Whitney U test. Categorical variables were indicated as number (%) and compared through Fischer’s Exact test. P value below 0.05 was accepted as statistical significance. Receiver operating characteristic (ROC) analysis was performed to detect the sensitivity and specificity of the test.

## RESULTS

**Figure 2:**
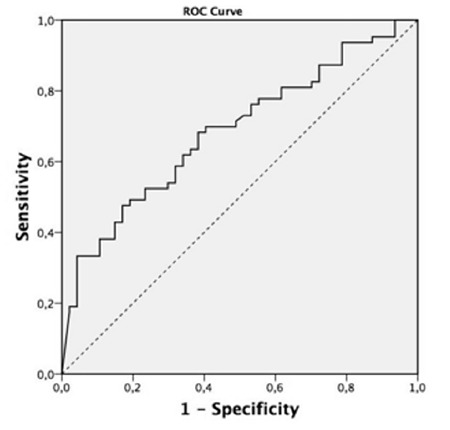
Receiver operating characteristic of increasing trend of NT-pro BNP in predicting mortality. AUC=0.84 (p<0.0001, 95% CI: 0.75-0.93) at cut-off value >5543 (sensitivity: 80%; specificity: 79%, PPV: 0.53, and NPV: 0.93). *AUC: area under curve; CI: confidence interval; ROC: receiver operating characteristic; NPV: negative predictive values; NT-pro BNP: N-terminal pro brain natriuretic peptide; PPV: positive predictive values*

**Figure 3:**
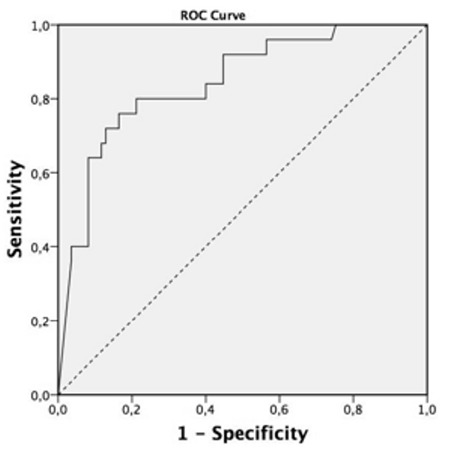
Receiver operating characteristic of increasing trend of NT-pro BNP in predicting invasive mechanical ventilatory need. AUC=0.68 (p=0.001, 95% CI: 0.8-0.78). *AUC: area under curve; CI: confidence interval; invasive mechanical ventilatory: invasive mechanical ventilatory; NT-pro BNP: N-terminal pro brain natriuretic peptide; ROC: receiver operating characteristic*

A total of 110 patients (75 males) from 1228 patients were included in the study. Patients’ demographic data are shown in Table 1. The median age was 69 (61-76) years, and the median APACHE II score was 19 (15-23). The most frequent comorbitidies were diabetes mellitus (n=18, 16.3%) and hypertension (n=9, 8.18%). Noninvasive mechanical ventilatory therapy was initiated in 69 patients due to type 2 respiratory failure. The noninvasive mechanical ventilatory success rate was 68.11%, and the median NT-pro BNP level was lower in cases with noninvasive mechanical ventilatory success than those with noninvasive mechanical ventilatory failure (2004 pg/mL vs. 3977 pg/mL, p=0.05). No differences in pH, pCO_2_, and PaO_2_/FiO_2_ ratio was found between patients with successful and failed noninvasive mechanical ventilatory. Invasive mechanical ventilatory was applied to 63 patients (41 due to noninvasive mechanical ventilatory failure). No differences in age, APACHE II score, pH, and PaO_2_/FiO_2_ ratio was found. Patients who required invasive mechanical ventilatory support had significantly higher NT-pro BNP levels (4740 pg/mL vs. 3004 pg/mL, p=0.001) than patients who did not. The NT-pro BNP levels in patients with weaning success were significantly lower than those in patients who failed weaning [2626 (964-5893) pg/mL vs. 11530 (3214-35000) pg/mL p=0.001]. Patient characteristics according to the trends of NT-pro BNP during ICU stay are shown in Table 2. Patients with an increasing trend of NT-pro BNP levels during ICU stay had significantly higher mortality rate (59% vs. 23%, p=0.015) than patients with a decreasing trend. Based on the ROC analysis, the increasing trend of NT-pro BNP levels during ICU stay was predictive of mortality with area under curve (AUC) of 0.84 (p<0.0001, 95% CI: 0.75-0.93) at cut-off value > 5543 (sensitivity: 80%; specificity: 79%, PPV: 0.53, and NPV: 0.93) (Figure 2). ROC analysis also predicted invasive mechanical ventilatory need with AUC of 0.68 (p=0.001, 95% CI: 0.8-0.78) with increasing trend in NT-pro BNP levels during ICU stay (Figure 3).

## DISCUSSION

This study shows that increasing NT-pro BNP levels during ICU stay may be a prognostic factor for ICU mortality and invasive mechanical ventilatory support requirement of patients with AECOPD.

Acute exacerbation affects the quality of life and prognosis of patients with COPD. A more severe disease, low FEV_1_, functional dyspnea, advanced age, presence of cardiovascular comorbidities, low body mass index, and low physical activity were identified as risk factors for exacerbation requiring hospitalization in patients with COPD (9,10). Plasma NT-pro BNP levels in patients with stable COPD were reported to be significantly higher than those in healthy subjects. Plasma NT-pro BNP levels were also reported to be significantly higher during AECOPD than those during stable disease (11,12,13,14). Cardiac causes are the most common causes of death in patients with COPD rather than respiratory complications (11,15). Elevated NT-pro BNP levels were also observed in patients with AECOPD without primary cardiac abnormalities, and AECOPD is thought to originate from both sides of the heart. Cor pulmonale, secondary pulmonary hypertension, and hypoxemia represent important stimuli for the release of NT-pro BNP from the right side of the heart (14,16). In our study, patients did not have a history or physical findings of primary cardiac failure. In some patients with COPD, air trapping and hyperinflation of the lung may be related to a decreased cardiac function and may lead to an increased level of NT-pro BNP (11,17). In hypercapnic respiratory failure prior to noninvasive mechanical ventilatory treatment, an association between high NT-pro BNP and high paCO_2_ levels were found. Previous works also reported significant reduction in NT-pro BNP and paCO_2_ levels after noninvasive mechanical ventilatory treatment (15,18,19). In the present study, NT-pro BNP levels were not correlated with paCO_2_ levels neither in failed noninvasive mechanical ventilatory nor successful noninvasive mechanical ventilatory cases. Elevated NT-pro BNP levels were found to be correlated with infection, organ failure, and sepsis in ICU patients and may be relevant to the severity of disease; hence, elevated NT-pro BNP levels could be used as an independent predictor of mortality, need for intubation, and invasive mechanical ventilatory application (11,15,20,21). We also found significant correlation between invasive mechanical ventilatory requirement and median NT-pro BNP levels. Moreover, increased levels of NT-pro BNP were correlated with ICU mortality rate. Previously unrecognized heart failure was present in 20% of patients with COPD (22). The measurement of NT-pro BNP levels is helpful in the diagnosis of heart dysfunction in patients with COPD especially during acute exacerbation (12,18). Previously unknown or new onset heart failure could be associated with weaning difficulties arising from mechanical ventilatory in patients with COPD. Scholars have increasingly focused on serial measurement of NT-pro BNP levels in patients with COPD. Elevated NT-pro BNP levels before or during spontaneous breathing trials (SBT) could be a predictor of weaning failure (23,24). Additionally, many studies have pointed out that higher levels of NT-pro BNP were significantly associated with in-hospital- (2), early- (30 days) (4), and long term- (1 year) mortalities (7,24). Measuring changes in the percentage of NT-pro BNP levels during SBT may help predict weaning outcomes (25). In the present study, elevated NT-pro BNP levels were strongly associated with invasive mechanical ventilatory need and mortality. Increased levels of NT-pro BNP might be related to the worsening of cardiorespiratory function resulting in invasive mechanical ventilatory requirement.

Our study presents several limitations. First, we used a retrospective design and included patients from a single center; as such, the generalization of the results is difficult. Second, even though patients who had primary cardiac diseases were not included in this study, we did not have the chance to perform advanced cardiac evaluation techniques, such as echocardiography. NT-pro BNP levels were measured and monitored randomly according to the clinical status of the patients.

Measurement of NT-pro BNP levels and monitoring their trend during ICU stay might be valuable tools for predicting invasive mechanical ventilatory need and ICU mortality in patients with AECOPD. Further studies are needed to test the reliability of this biomarker for the prognosis of patients in ICU.

## Figures and Tables

**Table 1:**
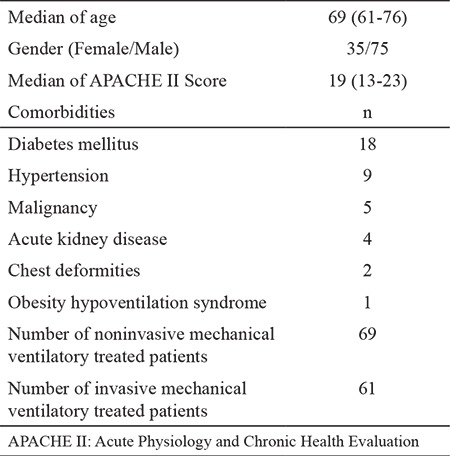
Patient’s demographic data

**Table 2:**
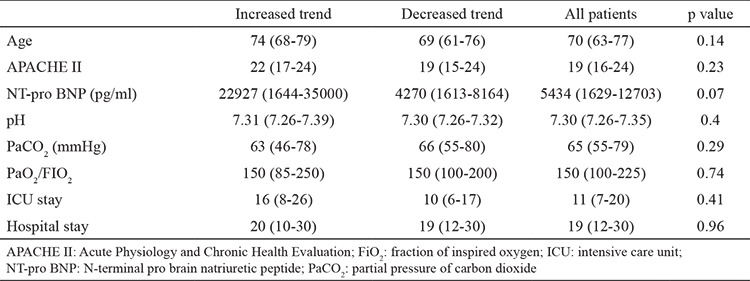
Characteristics of patients according to the trend of NT-pro BNP during ICU stay
